# Quadruplet regimen for newly diagnosed multiple myeloma is effective in the standard-risk subgroup but not in the high-risk subgroup

**DOI:** 10.3389/fphar.2024.1398879

**Published:** 2024-05-09

**Authors:** Jianghua Ding, Shengping Gong

**Affiliations:** ^1^ Department of Hematology and Oncology, Jiujiang University Affiliated Hospital, Jiujiang, China; ^2^ Department of Oncology, The First Affiliated Hospital of Ningbo University, Ningbo, China

**Keywords:** quadruplet regimen, newly-diagnosed, multiple myeloma, standard risk, high risk

## 1 Introduction

Multiple myeloma (MM) is the second most common hematologic cancer, with an estimated 35,730 new cases and 12,590 deaths annually in the United States (Siegel et al., 2023). With the introduction of new drugs, the number of drug combinations for MM treatment is constantly increasing. The 5-year overall survival (OS) rate for newly diagnosed multiple myeloma (NDMM) is 48.5% (Rajkumar, 2022). The National Comprehensive Cancer Network guideline (Ver. 2024.1) recommends triplet regimens [such as bortezomib + lenalidomide + dexamethasone (VRd) and daratumumab + lenalidomide + dexamethasone (DRd)] as the initial treatment for MM. Evidence has shown that for NDMM, triplet regimens achieve better efficacy than two-drug regimens (Derman et al., 2022; Yang et al., 2022). Currently, the triplet regimen has become the standard first-line therapy for NDMM. Recent clinical studies have explored the efficacy of quadruplet regimens in treating patients with NDMM (Table 1). In addition to studying the three categories of agents, namely, immunomodulatory drug, proteasome inhibitor, and steroid, efforts have also been devoted to study the anti-CD38 (daratumumab and isatuximab) and anti-SLAMF7 antibodies (elotuzumab). This opens a new possibility for analyzing the efficacy of quadruplet therapy as an alternative first-line treatment for NDMM. Based on concomitant cytogenetic abnormalities, NDMM patients can be classified into standard-risk (SR) and high-risk (HiR) subgroups (Rajkumar, 2022). This study aims to answer the following question: Is the quadruplet regimen better than the triplet regimen for treating patients with NDMM?

**TABLE 1:** Summary of prospective clinical trials about quadruplet treatment for NDMM patients.

Clinical study	Author/Year	Regimen	Sample size		Clinical outcomes			3/4 TRAE
					ORR	PFS (months)	OS (months)	
ALCYONE	Mateos M, 2018	D-VMP vs. VMP	350 vs. 356		≥CR: 46% vs. 25% (*p* < 0.001) MRD negative: 28% vs.7% (*p* < 0.001)	36.4 vs. 19.3 ms HR = 0.42 (0.34, 0.51)	36 m-OS: 78% vs. 67.9% HR = 0.60 (0.46, 0.80)	Neutropenia: 40% vs. 39% Infection: 22% vs. 15%
			SR	261 vs. 257	≥CR: 48% vs. 26% (*p* < 0.0001)	NE vs. 17.4 ms HR = 0.39 (0.28, 0.55)	—	—
			HiR	53 vs. 45	≥CR: 42% vs. 24% (*p* = 0.0764)	18.0 vs. 18.1 ms HR = 0.78 (0.43, 1.43)	HR = 0.91 (0.5, 1.65)	—
CASSIOPEIA	Moreau P, 2019	D-VRd vs. VRd	543 vs. 542		≥sCR: 29% vs. 20% OR = 1.60 (1.21, 2.12) MRD negative: 64% vs. 44% (*p* < 0.001)	NR vs. 46.7 ms HR = 0.49 (0.38, 0.62)	—	Lymphopenia: 4% vs. 2%
			SR	460 vs. 454	sCR: 30% vs. 19% OR = 1.82 (1.34, 2.48)	HR = 0.41 (0.26, 0.62)	—	—
			HiR	82 vs. 86	sCR: 24% vs. 28% OR = 0.83 (0.42, 1.66)	HR = 0.67 (0.35, 1.30)	—	
GRIFFIN	Voorhees P, 2020	D-VRd vs. VRd	104 vs. 103		sCR: 67% vs. 48% ≥VGPR: 90.9% vs. 73.2% MRD negative: 51.0% vs. 20.4%	24-ms: 95.8% vs. 89.8%. 48-ms: 87.2% vs. 70%	—	Neutropenia: 46% vs. 23% Infection: 23.2% vs. 21.6% VTE: 9.1% vs. 14.7%
			SR	82 vs. 83	MRD negative: 54.9% vs. 20.5% OR = 4.72 (2.37, 9.40)	—	—	—
			HiR	16 vs. 14	MRD negative: 37.5% vs. 28.6% OR = 1.5 (0.32, 6.99)	—	—	—
OCTANS	Fu W, 2023	D-VMP vs. VMP	146 vs. 74		≥VGPR: 74% vs. 43.2% OR = 3.57 (1.99, 6.43) MRD negative: 29.5% vs. 6.8% OR = 6.19 (2.29, 16.75)	NR vs. 18.2 ms HR = 0.43 (0.24, 0.77) 12-ms: 84.2% vs. 64.6%	—	Thrombocytopenia: 46.5% vs. 45.1% Neutropenia: 39.6% vs. 50.7% Pneumonia: 27.8% vs. 14.1%
			SR	117 vs. 54	≥VGPR: 74.4% vs. 40.7% OR = 4.22 (2.13, 8.35)	HR = 0.45 (0.24, 0.84)	—	—
			HiR	28 vs. 20	≥VGPR: 75% vs. 50% OR = 3.0 (0.88, 10.21)	HR = 0.34 (0.09, 1.32)	—	—
SWOG-1211	Usmani S, 2022	Elo-VRd vs. VRd	49 vs. 54		—	29 vs. 34 ms HR = 1.11 (0.82, 1.49)	NR vs. 68 ms HR = 0.85 (0.59, 1.23)	Infections: 16% vs. 8% Sensory neuropathy: 13% vs. 8%
			1q21	48	—	31 vs. 37 ms HR = 1.48 (0.95, 2.31)	61 vs. 68 ms HR = 1.23 (0.72, 2.10)	—
			17p-	40	—	41 vs. 30 ms HR = 0.98 (0.60, 1.58)	NR vs. 72 ms HR = 0.77 (0.40, 1.48)	—
GMMG-HD7	Goldschmidt H, 2022	Isa-VRd vs. VRd	331 vs. 329		MRD negative: 50% vs. 36% OR = 1.82 (1.33, 2.48)	—	—	Neutropenia: 23% vs. 7% Infection: 12% vs. 10%
			SR	254 vs. 234	MRD negative: OR = 1.88 (1.30, 2.72)	—	—	—
			HiR	58 vs. 66	MRD negative: OR = 1.81 (0.89, 3.72)	—	—	—
Myeloma XI+	Jackson G, 2021	KRdc vs. Rdc or Tdc	530 vs. 526		≥VGPR: 82.3% vs. 58.9% MRD negative: 50.9% vs.12.7%	NR vs. 36.2 ms HR = 0.63 (0.51, 0.76) 36 ms: 64.5% vs. 50.3%	—	Anemia: 10% vs. 4.8%; Neutropenia: 11.5 vs. 8.9%-
			SR	101 vs. 103	-	NR vs. 37 ms HR = 0.62 (0.39, 0.98)	—	—
			HiR	81 vs. 60	—	NR vs. 37 ms HR = 0.68 (0.40, 1.14)	—	—
			UHR	22 vs. 16	—	36 vs. 20 ms HR = 0.50 (0.20, 1.25)	—	—

Abbreviation: *ORR*, objective response rate; *PFS*, progression-free survival; *OS*, overall survival; *TRAE*, treat-related adverse effect; SR, standard-risk; HiR, high-risk; UHR: ultra high risk; *D-VMP*, daratumumab, bortezomib, melphalan, and prednisone; *VMP*, bortezomib, melphalan, and prednisone; *CR*, complete remission; *MRD*, minimal residual disease; *VGRP*, very good partial remission; *D-VRd*, daratumumab, bortezomib, lenalidomide, dexamethasone; *sCR*, stringent complete remission; *VRd,* bortezomib, lenalidomide, dexamethasone; *NR*, not reached; *Elo-VRd*, elotuzumab, bortezomib, lenalidomide, dexamethasone; *Isa-VRd*: isatuximab, bortezomib, lenalidomide, dexamethasone; KRdc, carfilzomib, lenalidomide, dexamethasone, and cyclophosphamide; Rdc, lenalidomide, dexamethasone, and cyclophosphamide; Tdc, thalidomide, dexamethasone, and cyclophosphamide.

## 2 Controversy about the use of quadruplet regimens for treating SR NDMM patients

Approximately 80% of NDMM patients belong to the SR subgroup, with a median OS ranging from 8 to 10 years (Goel et al., 2022). Recent studies have explored the efficacy of quadruplet regimens containing anti-CD38 antibodies in treating NDMM patients. As shown in Table 1, except for the SWOG-1211 study (Usmani et al., 2022), the other six prospective clinical studies, namely, ALCYONE (Mateos et al., 2020), CASSIOPEIA (Moreau et al., 2021), GRIFFIN (Voorhees et al., 2023), OCTANS (Fu et al., 2023), GMMG-HD7 (Goldschmidt et al., 2022), and Myeloma XI+ (Jackson et al., 2021), revealed that for SR NDMM patients, quadruplet regimens achieved better overall response rates (ORRs), longer progression-free survival (PFS), and higher rates of minimal residual disease (MRD) negativity than corresponding triplet regimens. Of note, the status of MRD represents the depth of post-therapeutic remission and serves as an independent prognostic factor for NDMM patients (San-Miguel et al., 2022). It seems logical that increased MRD negativity by quadruplet regimens containing anti-CD38 antibodies will result in prolonged OS of SR NDMM patients. Therefore, it is rational to use quadruplet regimens containing anti-CD38 antibodies for SR NDMM patients due to their favorable efficacy compared with that of the triplet regimen (VRd).

However, as shown in Table 1, the data on OS of the five studies are not yet available, except for the favorable OS benefit in the ALCYONE study (Mateos et al., 2020). On the contrary, the SWOG-1211 study reported no survival advantage from the quadruplet regimen of Elo-VRd over the VRd regimen (Usmani et al., 2022). Furthermore, an issue that cannot be ignored is the incremental cost-effectiveness ratio (ICER). According to a recent investigation, daratumumab + bortezomib + melphalan + predisone (D-VMP) vs. bortezomib + melphalan + predisone (VMP) has a 90.8% probability of being cost-effective at the $150,000/quality-adjusted life year willingness-to-pay threshold (Zeng et al., 2021). Compared with VMP, D-VMP may exceed the commonly accepted values of ICER in patients with NDMM in China. Thus, it is necessary to consider the cost-effectiveness of the quadruplet regimen for SR NDMM patients, especially in developing countries.

## 3 Controversy about the use of quadruplet regimens for treating HiR NDMM patients

Nearly 20% of patients with NDMM belong to the HiR subgroup, with features including del (17p), t (4:14), t (14:16), t (14; 20), TP53 mutation, R-ISS stage III, gain (1q) (identified using cytogenetic/fluorescence *in situ* hybridization analysis), high plasma cell S-phase, and HiR signature of gene expression profiling. This group also contains an ultra-high risk (UHR, i.e., double/triple-hit) subgroup. Compared with the SR NDMM subgroup, the HiR NDMM subgroup has a predicted OS of less than 3 years (Zamagni et al., 2022). According to the meta-analysis by Giri et al. (2020), incorporating daratumumab into primary regimens may improve PFS [pooled hazard ratio (HR) = 0.67, 95% confidence interval (CI): 0.47–0.95] in HiR NDMM patients. However, in the studies of COSSIPEIA and ALCYONE (Table 1), statistically significant benefits were not yet seen with the addition of daratumumab as a fourth drug to a triple-drug regimen in newly-diagnosed HiRMM. The MAIA study compared the efficacy of regimens DRd and Rd (HR = 0.53, 95% CI: 0.43–0.66, *p* < 0.001; HR = 0.68, 95% CI: 0.53–0.86, *p* = 0.0013) (Facon et al., 2021; Facon et al., 2023). Furthermore, the small sample size (only 317 in total) might have reduced the statistical power of the meta-analysis in cases of HiR NDMM. According to the results above, Mohyuddin et al. (2021) held that it is prudent to routinely use a daratumumab-based regimen for HiR NDMM patients.

As described in Table 1, in six studies, all of the subgroup analyses of HiR NDMM revealed that compared with triplet regimens, quadruplet schemes failed to yield a statistically favorable clinical outcome, including ORR, PFS, and MRD-negative rate. At this point, caution should be exercised when choosing a quadruplet regimen as the first-line treatment for HiR NDMM patients until we have OS data to justify additional adverse effects and potential long-term costs. The Myeloma XI + study found that UHR NDMM patients on the KRdc quadruplet regimen had a longer PFS than those on the Rdc or Tdc triple regimen but without any statistical difference (Jackson et al., 2021). These results strongly indicate that it is premature to recommend the use of quadruplet regimens for HiR NDMM patients.

## 4 Expert opinion

A network meta-analysis by Facon et al. (2022) showed that daratumumab-based regimens, including D-Rd, D-VMP, and VRd, had the highest probabilities of being more effective than Rd continuous in terms of PFS (HR: D-Rd, 0.53; D-VMP, 0.57; VRd, 0.77) and OS (HR: D-Rd, 0.68; VRd, 0.77; D-VMP, 0.78) for NDMM patients. Among them, D-Rd ranked first as the most effective treatment in terms of PFS and OS. Given the excellent efficacy of triplet regimens such as D-Rd and VRd, we recommend careful consideration when choosing a quadruplet regimen as the first-line treatment for patients with NDMM. For the SR subgroup, the use of anti-CD38 antibody-based quadruplet treatment appears to be more effective than the triplet regimen. However, cost-effectiveness should be considered, particularly in developing countries. For the HiR subgroup, based on currently available evidence, the quadruplet treatment appears to be ineffective, as no superiority in efficacy has been found compared with that of the triplet regimen.
